# Semantic-based memory-encoding strategy and cognitive stimulation in enhancing cognitive function and daily task performance for older adults with mild cognitive impairment: A pilot non-randomised cluster controlled trial

**DOI:** 10.1371/journal.pone.0283449

**Published:** 2023-03-27

**Authors:** Hannah Mraz, Choy-Ying Tong, Karen P. Y. Liu

**Affiliations:** 1 School of Health Sciences, Western Sydney University, Sydney, Australia; 2 Christian Family Service Centre, Kwun Tong, Hong Kong; 3 Translational Health Research Institute, Western Sydney University, Sydney, Australia; 4 Department of Rehabilitation Sciences, The Hong Kong Polytechnic University, Hung Hom, Hong Kong; Alanya Alaaddin Keykubat University: Alanya Alaaddin Keykubat Universitesi, TURKEY

## Abstract

**Aim:**

To investigate the effectiveness of a semantic-based memory-encoding strategy intervention and cognitive stimulation that enhances function for older adults with mild cognitive impairment.

**Methods:**

A two-armed single-blind non-randomised cluster controlled trial was conducted. Participants in two centres were allocated to the semantic-based memory-encoding experimental group and those in the other two centres received cognitive stimulation. In both groups, one centre- or community-based session and one home-based session were provided weekly for 10 weeks. Outcome measures included attention, memory and general cognitive function (Word List Memory and Word List Recall of the Consortium to Establish a Registry for the Alzheimer’s disease, Digit Span Forwards and Backwards and the Cognistat), and daily task performance (Disability Assessment for Dementia and Lawton Instrumental Activities of Daily Living Scale). They were administered pre- and post-intervention.

**Results:**

Thirty-nine participants completed the study. No significant differences were revealed in the demographic or baseline data. The experimental group showed significant improvements in daily task performance (Disability Assessment for Dementia; *p* = 0.003), memory outcomes (Word List Recall; *p* < 0.001), general cognitive function (Cognistat subtests of Memory and Similarity; *ps* = 0.002 and < 0.001). The cognitive stimulation control group did not show any significant improvement in the measures. Between-group analysis showed significant differences in favour of the experimental group for the outcome measures of the Word List Recall and Cognistat Similarity subtest (*ps* < 0.001).

**Conclusions:**

This study shows that the semantic-based memory-encoding strategy is more superior than cognitive stimulation with improvements in attention, memory, general cognitive function and daily task performance for people with a mild cognitive impairment.

**Trial registration:**

ClinicalTrials.gov Protocol Registration and Results System (NCT02953964).

## Introduction

The world is experiencing the growth of older adults in the population. For example, Australia’s older population (75 years and over) is expected to increase by four million from 2012 to 2060, with the baby-boomer generation entering old age [1]. Nearly 20 per cent of adults aged over 65 years are affected by mild cognitive impairment and up to 70 per cent of them will go on to develop a type of dementia [2, 3]. Research supports a prevention strategy before the older adults develop dementia and lose their ability to perform daily tasks [4–6]. Effective early-intervention strategies for older adults with mild cognitive impairment to remediate and delay the deterioration of daily functioning are essential. A recent systematic review reported two types of cognitive interventions, including cognitive stimulation and cognitive training, that improve or remediate cognitive function [7].

Cognitive stimulation is used in activities that have been designed to increase cognition and social functioning in a non-specific manner, such as reading, playing chess, drawing and painting [7, 8]. Cognitive stimulation activities target a specific cortical area that is related to the cognitive domain being investigated and boost cognitive reserves to prevent cognitive decline [9, 10]. Cognitive training uses practising cognitive tasks that aim to improve or maintain cognitive functions [11]. Training that uses applied memory strategies with repetitive cognitive exercises is one example [4, 12].

Among the various memory strategies, the semantic-based memory-encoding strategy uses an association format to encode by linking information relevant to an individual’s context [4]. Semantic processing can lead to the greater probability of an item being encoded into long-term memory [6, 13]. Previous evidence regarding the use of this strategy has shown positive results relating to memory and general cognitive abilities for healthy older adults [5]. Cansino, Trejo-Morales [14], Kuo, Liu [6] have commented that using meaningful tasks or materials with repeated practice to enhance elaboration might promote the use of semantic-based memory-encoding strategy in older adults. Simon, Yokomizo [2] suggest that despite cognitive deficits, people with mild cognitive impairment have some preserved ability to learn new information and adapt behaviours that support the implementation of the semantic-based memory-encoding strategy. It is, therefore, worthwhile exploring the use of this strategy in older adults at the stage of mild cognitive impairment, which is the earliest stage of cognitive impairment that shows clinical signs.

This study investigated the use of a semantic-based memory-encoding strategy intervention, compared to cognitive stimulation, on attention, memory, general cognition and the daily performance of tasks by older adults with mild cognitive impairment using a pilot non-randomised cluster controlled study design. This study could provide evidence for early intervention to improve cognition and daily-task performance in everyday life for older adults with mild cognitive impairment.

## Methods

### Study design

This was a pilot study that adopted a single-blind non-randomised cluster controlled trial design. Ethics approval was provided by the local ethics commitee. The study was registered with the ClinicalTrials.gov Protocol Registration and Results System (NCT02953964).

### Participants and recruitment

Participants were recruited from four community elderly care centres affiliated with a non-government organisation in Hong Kong Special Administrative Region. Before the recruitment of the participants, four centres were assigned to either the semantic-based memory-encoding strategy (SmeS) experimental group or the cognitive stimulation (CS) control group. Potential participants were then identified by staff at the centres using the following selection criteria. Once the potential participants were identified, the research team approached the participants and their family members to explain the project and obtain written informed consent.

The selected participants were aged 60 or over and had no previous psychiatric or memory disorder history or other neurological illness, were able to communicate effectively, had voluntarily provided consent to participate in the study and had a family member who could participate in the study. The participants had a Mini-Mental State Examination score greater than or equal to 21, a diagnosis of mild cognitive impairment according to the Petersen criteria, which includes memory complaints corroborated by an informant, essentially preserved general cognitive function, largely intact functional activities, and no diagnosis of dementia as shown by a clinical dementia rating of 0.5 [15]. The participants did not show signs of depression with scores below 9 out of 30 on the Geriatric Depression Scale.

**Fig 1:**
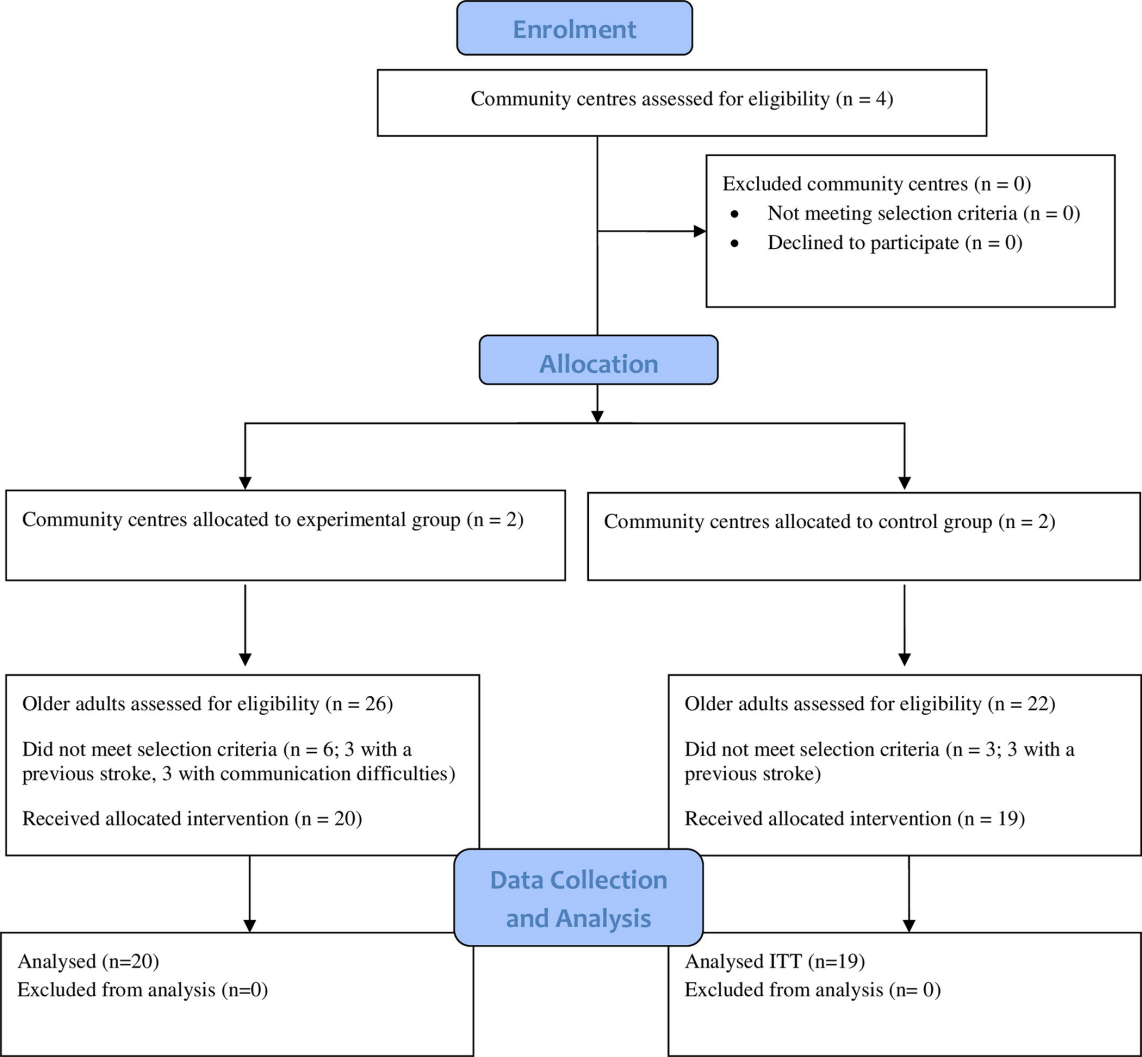
Consort flow diagram.

A researcher blinded to the group allocation was responsible for obtaining written consent. The participant allocation process is shown in the flowchart in Fig 1.

### Intervention

A 10-week intervention program was run for the two participant groups–the SmeS experimental group and the CS control group. Each weekly group session consisted of six to eight participants and ran for 90 minutes. For both groups, a follow-up home training program with the participants and their caregivers was also run by healthcare workers and volunteers once a week for 30 minutes.

**Fig 2:**
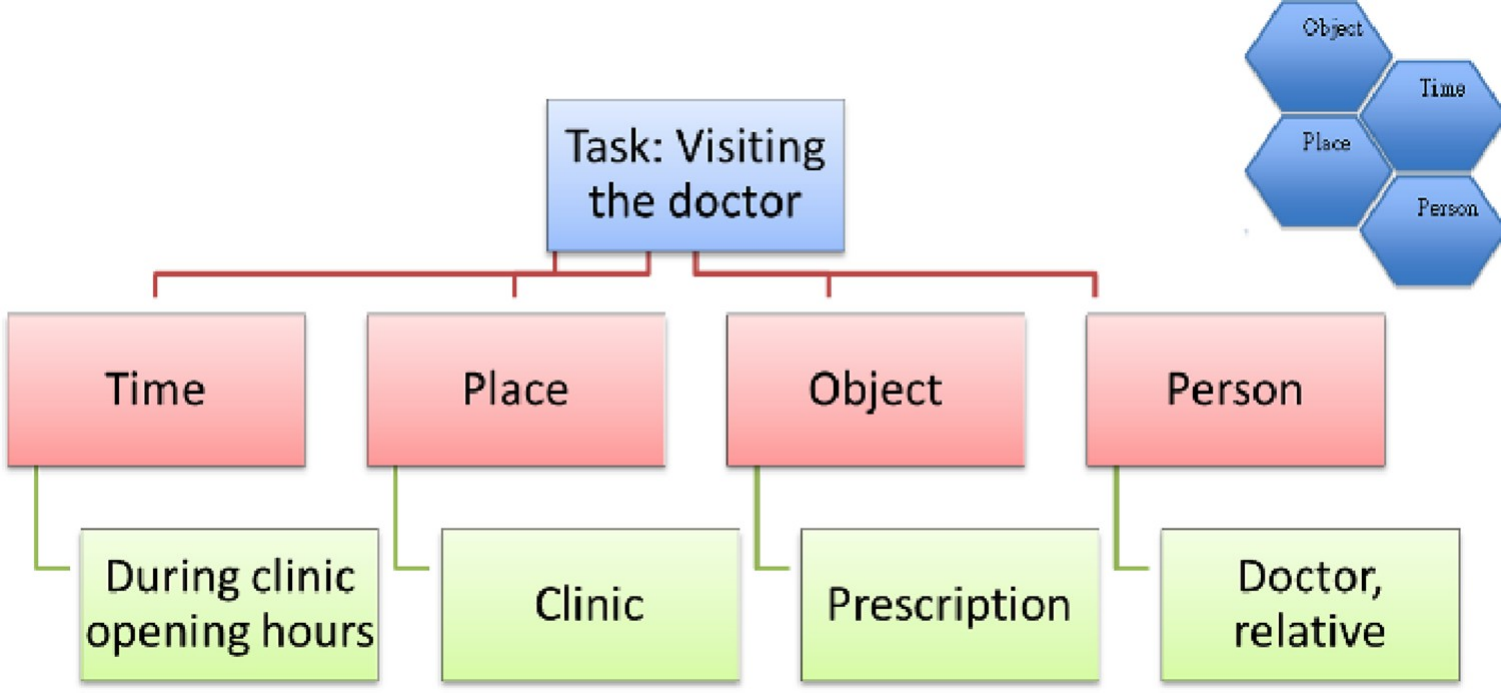
Example using the honeycomb concept.

The SmeS experimental group consisted of 10 centre-based group sessions. Each session was run by an occupational therapist and two additional facilitators who were independent of the participant recruitment and data collection processes. The participants were asked to encode the steps of a task by forming an association with the steps and sequence. They were trained in the use of the chunking association method and honeycomb concept and performed different tasks each week, implementing this encoding strategy [4]. A chunk is a collection of items where each item is strongly associated with each of the others [16]. The chunking association method breaks down a task into smaller parts, or steps, and helps with the encoding and retrieval of information. The honeycomb concept enables the steps to form a story, which is associated with, and relates to, place, time, characters, problem and solution and leads to the story being verbalised (Fig 2 and S1 Appendix). The participants then completed the task in the centre environment.

The CS control group programme consisted of eight centre-based sessions that trained the participants’ visual attention and memory, their auditory attention and memory, and then the application of these in the participants’ daily activities. Two community-based sessions were also provided, where the participants were given the opportunity to apply the training practically in their daily lives. Each session was run by an occupational therapist and two additional facilitators.

Each session for both the SmeS experimental and CS control groups consisted of an introduction to the session, specific strategies or games used in the respective group, the completion of two to three tasks, a game, and revision with time for questions. The same 15 common daily tasks were performed in both groups, including:handling and folding laundry; making the bed; making tea; washing dishes; using a telephone; visiting a doctor; taking medication; sweeping; meal preparation; cleaning after a meal; cutting fruit; going to the park; shopping; and going to the canteen. Lim, Liu [4] support the use of daily tasks as the context for teaching memory-encoding strategies. Practical application of the tasks is incorporated in the learning to promote the understanding of use in daily life.

### Data collection

The participants were assessed for baseline data and then assessed for changes after the intervention. The assessors were blinded to the group allocation of the participants.

### Outcome measures

The outcome measures used included daily task performance as the primary outcome measure, and attention and memory performance, general cognitive function as the secondary outcome measures. All assessments were administered in a random order for each participant.

### Daily task performance

The Disability Assessment for Dementia (DAD) is a 46-item survey, completed by the caregiver, that evaluates the level of independence in instrumental and personal daily tasks over the previous two weeks for a person who has dementia or cognitive impairment [17]. The DAD has excellent inter-examiner reliability and test-retest reliability, a high degree of internal consistency and established criterion validity [17]. The DAD is considered to be the gold standard for clinical trials that measure functional ability [18].

The Lawton Instrumental Activities of Daily Living Scale (IADLS) assesses the ability to independently perform eight domains of instrumental daily tasks. The self-reported format was used in this study. Items are rated on a three-point scale (*unable*, *with help*, *independent*) [19]. Reliability, inter-rater reliability and validity were all established for this test as a baseline and comparative test [20].

### Attention and memory performance

The Consortium to Establish a Registry for Alzheimer’s Disease–Neuropsychological Test Battery (CERAD–NAB) provides a profile on cognitive function by assessing cognitive deficits such as memory impairment, disorientation, language and dyspraxia [21]. Evidence strongly supports the validity and usefulness of the CERAD for the early detection of mild cognitive impairment [22]. In this study, the Word List Memory (J4) subtest and Word List Recall (J6) subtest were used.

The Wechsler’s Digit Span Test–consisting of the repetition of digits forward (Digit Span Forward [DSF]) and backwards (Digit Span Backward [DSB])–measures immediate verbal recall, attentional capacity and working memory [23]. The test, particularly, DSB, can predict the presence of mild cognitive impairment; however, age, gender and education do have an impact on performance [24].

### General cognitive function

The Neurobehavioural Cognitive Status Examination (Cognistat) is a measure that provides independent scores for memory, language, visuospatial function, arithmetic and verbal reasoning [25]. Evidence indicates that it is sensitive to normal ageing [26]. The Cognistat has good validity for detecting cognitive impairment [27].

### Statistical analysis

Descriptive statistics were reported on the demographic data and all outcome measures. Demographic and baseline data were compared between the two groups using an independent t-test or a Chi-square test for interval and categorical data to assess for any potential disparities between the two groups. To evaluate the effectiveness of the interventions, the Wilcoxon Signed Rank Test was used for within-group data (pre- and post-intervention assessments) and the Mann-Whitney Test was used for between-group comparison (two groups). The Bonferroni correction was adopted to account for multiple comparison. Significance levels were set at smaller than or equal to 0.003. Data from the pre- and post-intervention assessments was analysed using SPSS software.

## Results

A total of 39 participants (16 men and 23 women; mean age– 79.67 years; age range– 63 to 91 years) completed the trial– 20 in the SmeS experimental group and 19 in the CS control group. They had all been diagnosed with mild cognitive impairment according to Petersen’s criteria [15] and had received a clinical dementia rating of 0.5 according to the inclusion criteria. The Mini-Mental State Examination scores revealed no difference between the two groups. In addition, no significant differences were found in the demographic data relating to age, gender or years of education and the baseline data between the two groups (see Table 1).

**Table 1:** Demographic data and mini-mental state examination scores.

	Semantic-based memory-encoding strategy group (n = 20)	Cognitive stimulation group (n = 19)
Age (years): mean ± SD	80.85 ± 5.85	78.42 ± 9.47
Gender (female): number, percentage	12, 60%	11, 58%
Education (years): mean ± SD	1.6 ± 1.88	2.11 ± 2.73
Mini-Mental State Examination: mean ± SD	23.70 ± 1.75	23.63 ± 1.67

Results from the within-group analyses showed significant improvement in the SmeS experimental group across a few outcome measures when comparing pre- and post-intervention scores. No significant improvement was found in any measures in the CS control group. For daily task performance, participants in the SmeS experimental group showed significant improvement in the DAD (*p* = 0.003) (see Table 2). For memory outcomes, the participants in the SmeS experimental group showed significant improvement in CERAD-NAB Word List Recall (J6) (p < 0.001). For general cognitive function tested by the Cognistat, participants in the SmeS experimental group showed significant improvement in the subtests of Memory (*p* = 0.002) and Similarity (*p* < 0.001). Between-group analysis showed significant differences in favour of the SmeS experimental group in CERAD-NAB Word List Recall (J6) (*p* < 0.001) and Cognistat Similarity subtest (*p* < 0.001) (see Table 2).

**Table 2:** Pre- and post-intervention data.

		Semantic-based memory-encoding strategy group (*n* = 20)		Cognitive stimulation group (*n* = 19)		Between-group difference *p* value
		Pre-intervention	Post-intervention	Pre-intervention	Post-intervention	
Daily task performance	Disability Assessment for Dementia	68.21 ± 20.21	75.06 ± 18.39 ^#^	65.96 ± 19.05	70.21 ± 16.42	0.36
	Lawton Instrumental Activities of Daily Living	4.95 ± 2.21	4.90 ± 2.49	4.37 ± 2.22	5.00 ± 2.16	0.14
Attention and Memory	Word List Memory (J4)^a^	11.20 ± 7.16	12.95 ± 7.35	11.47 ± 6.28	11.74 ± 5.60	0.34
	Word List Recall (J6)^a^	2.35 ± 3.05	4.90 ± 3.35 ^#^	2.37 ± 2.12	2.58 ± 2.12	<0.001**
	Digit Span Forwards	4.80 ± 2.14	5.40 ± 1.57	5.21 ± 1.75	5.53 ± 2.39	0.32
	Digit Span Backwards	1.85 ± 0.81	2.35 ± 0.67	1.58 ± 0.77	1.68 ± 0.82	0.11
General cognitive function	Orientation^b^	10.20 ± 1.82	9.95 ± 2.44	9.63 ± 1.83	9.74 ± 1.94	0.87
	Attention^b^	6.25 ± 2.25	7.40 ± 1.70	7.68 ± 0.82	7.58 ± 1.43	0.08
	Comprehension^b^	5.10 ± 1.12	5.75 ± 0.79	5.53 ± 1.02	5.68 ± 0.67	0.09
	Repetition^b^	6.10 ± 3.57	6.00 ± 3.34	4.58 ± 3.58	4.42 ± 3.78	0.82
	Naming^b^	5.25 ± 2.59	6.80 ± 2.09	5.68 ± 2.19	6.42 ± 1.90	0.17
	Construction^b^	2.65 ± 2.58	2.60 ± 2.26	2.26 ± 2.45	2.50 ± 2.23	0.74
	Memory^b^	5.35 ± 4.15	7.65 ± 3.72 ^#^	5.53 ± 4.03	6.22 ± 3.47	0.14
	Calculation^b^	2.50 ± 1.24	2.95 ± 1.15	2.00 ± 1.11	2.05 ± 1.22	0.13
	Similaritiy^b^	2.55 ± 1.36	4.70 ± 1.26 ^#^	2.84 ± 1.71	3.16 ± 1.83	<0.001**
	Judgement^b^	4.30 ± 1.22	5.00 ± 0.46	4.63 ± 0.90	4.58 ± 1.02	0.28

^a^ subtext of the Consortium to Establish a Registry for the Alzheimer’s Disease–Neuropsychological Test Battery

^b^ subtest of the Cognistat

^#^ denotes *p* ≤ 0.003 for within-group differences.

** denotes *p* < 0.001for between-group differences.

## Discussion

The aim of this study was to determine the effectiveness of a semantic-based memory-encoding strategy (SmeS experimental group) compared to cognitive stimulation (CS control group), for improving daily task performance, attention, memory and general cognitive function for older adults with mild cognitive impairment. The results indicated that the participants in the SmeS experimental group showed improved performance in the areas of daily task performance, memory and general cognitive function, whereas the CS control group did not produce any significant improvement after the intervention. A comparison of the two groups revealed that the SmeS experimental group had outperformed the CS control group in the CERAD Word List Recall and the Cognistat Similarity subtest. The results of this study have provided preliminary evidence of the semantic-based memory-encoding strategy intervention and support its effectiveness for people with mild cognitive impairment.

The SmeS experimental group used chunking and the honeycomb method. When information is chunked together, memory span can increase significantly [28]. Chunking is a simple but effective tool and has been suggested as a main strategy for successful cognitive training programs [29]. Chunking helps break down information (into chunks) and facilitates the forming of association in a person’s life, when the honeycomb method is used, by giving context to the chunks of information. The participants related the information according to the object, time, place and person(s) that were relevant to their contexts. This association method that was used may explain the significant improvement in the Cognistat Similarity subtest.

The ability of the brain to learn and adapt has been successfully demonstrated for adults in later life with the use of memory training, even in small amounts [30]. There is the potential that the semantic-based memory-encoding strategy may improve the encoding and retrieval process and utilise areas of the brain that have not yet been affected by cognitive decline in older adults with mild cognitive impairment [31, 32]. The results of this study support previous evidence of the effectiveness of the chunking and association techniques in relation to memory and cognitive ability in various populations [29, 33]. It is also in line with the evidence from Craik and Rose [30] and Simon, Yokomizo [2] that supports the concept of neuroplasticity and the brain’s ability to learn new information in later life despite cognitive deficits.

The results showed that the SmeS experimental group had significant improvement across a few areas. The responses to the DAD survey showed the participants’ improved daily function. Improvements in memory had also been shown by the CERAD-NAB J6 and Memory subtest of the Cognistat. Improvements in general cognitive function were shown by the Cognistat subtests of Attention and Similarity. These improvements are associated with the use of the semantic memory. The semantic memory relates new information to what is already known (information in the semantic memory). Deficits in working memory are evident in people with mild cognitive impairment. However, due to the use of already-formed memories (semantic information), the semantic-based memory-encoding strategy helps to associate from previous life contexts and enhance the encoding of information [34].

Cognitive stimulation is a commonly used intervention that has been shown to benefit various cognitive domains in healthy individuals [35] and for people with mild cognitive impairment [35]. Although no significant difference was found before and after the intervention in all measures, cognitive stimulation may enhance performance in cognitive domains by targeting a specific cortical area [35]. Our CS control group intervention might have provided limitation stimulation in targeted area.

Comparing the results of the two groups, the similarities and differences in scores are evident. Although the SmeS experimental group improved sigificantlly in the DAD of daily task performance, no significant difference was found in both daily task performance measures between the two group. This can be attributed to the use of daily tasks in the implementation of the interventions. Alternatively, this could have been due to the group’s already-high baseline score (SmeS experimental group baseline mean score = 4.95, CS control group baseline mean score = 4.37), where the performance had already reached the plateau and therefore further improvement would not be reflected by the assessment. However, there was a significant difference in improvement when the scores form the CERAD-NAB Word List Recall and the Cognistat Similiarity subtest were compared, with the SmeS experimental group showing greater improvement. These discrepancies between improvement in memory and general cognitive function may be explained by the use of the semantic-based memory-encoding strategy breaking down the information and forming associations to information that was already known.

These results are important because they show great potential for the use of semantic-based memory-encoding strategy to be effective for people who have mild cognitive impairment. This study has added to the research evidence for the use of chunking and association as a semantic-based memory-encoding strategy to improve attention, memory, cognitive and daily task performance. When the strategy is used effectively, it could possibly delay the impact of cognitive decline on functional deterioration when the disease progresses, although further study would be required to verify this postulation. This study, combined with previous research, supports the benefits of intervening at the earliest possible stage of cognitive impairment [32]. As a follow-on study from that of Lim, Liu [4], where the use of cognitive stimulation, semantic-based memory-encoding and perceptual-based memory-encoding strategies was combined, this study has shown the effectiveness of the semantic-based memory-encoding strategy independently of other interventions.

This study was limited by the small sample size and the non-randomisation methods. The selection criteria for people with MCI and the determination of other diagnoses through the report of the community centre staff posed a limitation on participants’ selection. The results are less likely to be able to be generalised to the greater population. Second, the intervention time of 10 weeks and lack of long-term follow-up might have reduced understanding of the participants’ long-term maintenance of these cognitive and functional abilities. Thirdly, the outcome measures being conducted before and after the intervention might pose a learning effect on participants and affect the results. The use of self-reported daily task performance measures could not provide objective measures of the participants’ daily task performances. The study result was also limited by the lack of follow-up assessment to evaluate if the cognitive gains sustained with the semantic encoding strategy have been preserved. Finally, a slight difference in the demographic characteristics such as education level in the two groups might create a possible difference in the results although no significance was found.

Future research should focus on maintaining function over the long term and delaying the impact of cognitive decline due to mild cognitive impairment or dementia on daily functioning. In addition, future studies should have a larger sample size using a randomised method using objective, sensitive and mild cognitive impairment- or dementia-specific measures to allow for generalisability to the greater population. For example, a more sensitive word list test, such as California Verbal Learning Test, can be used to evaluate episodic functions [36]. The use of daily tasks, as in the current two intervention programs, is recommended as it is a practical and effective way to apply the use of memory strategies to daily life [4].

## Conclusions

The findings from this study suggest that the semantic-based memory-encoding strategy that uses chunking and association is a promising intervention for maintaining and even improving daily task performance, attention and memory when intervening at the mild cognitive impairment stage. Previous research has had a focus on the changes in memory and cognition after performing a cognitive training intervention. This study showed that the specific semantic-based memory-encoding strategy had a better outcome to enhance the cognitive function for older people with mild cognitive impairment necessary for maintaining their daily functioning.

Future studies looking at interventions for reducing cognitive limitations should focus on their relation to improvements or maintenance in functional performance for older adults with mild cognitive impairment. Due to people living longer, it is essential to implement interventions at the earliest possible stage to slow the deterioration of cognition and function in the ageing population.

## Supporting information

S1 Checklist (DOC)

S1 File (PDF)

S2 File (PDF)

S1 Appendix (DOCX)

S1 Data (CSV)
