# Supplementary material for: Semantic-based memory-encoding strategy and cognitive stimulation in enhancing cognitive function and daily task performance for older adults with mild cognitive impairment: A pilot non-randomised cluster controlled trial
Source: PLoS One. 2023 Mar 27;18(3):e0283449. doi: 10.1371/journal.pone.0283449 (PMC10042350; doi:10.1371/journal.pone.0283449)
Supplement: S1 File — (PDF) [file pone.0283449.s002.pdf]

ClinicalTrials.gov PRS
Protocol Registration and Results System

ClinicalTrials.gov Protocol Registration and Results System (PRS) Receipt
Release Date: November 1, 2016

ClinicalTrials.gov ID: NCT02953964

Study Identification

Unique Protocol ID: Memory encoding

Brief Title: Memory Encoding Strategies for People With Mild Cognitive Impairments

Official Title: Perceptual and Semantic Memory Encoding Strategies for People With Mild Cognitive Impairment

Secondary IDs:

Study Status

Record Verification: November 2016

Overall Status: Completed

Study Start: May 2010 []

Primary Completion: October 2015 [Actual]

Study Completion: October 2015 [Actual]

Sponsor/Collaborators

Sponsor: University of Western Sydney

Responsible Party: Principal Investigator
Investigator: Karen Liu [kliu]
Official Title: Associate Professor
Affiliation: University of Western Sydney

Collaborators:

Oversight

U.S. FDA-regulated Drug:

U.S. FDA-regulated Device:

U.S. FDA IND/IDE: No

Human Subjects Review: Board Status: Approved
Approval Number: 11/03/2015
Board Name: Western Sydney University human Research Ethics Committee
Board Affiliation: Western Sydney University
Phone:
Email: humanethics@westernsydney.edu.au
Address:

Western Sydney University

Locked Bag 1797
Penrith NSW 2751
Australia

Data Monitoring: No

FDA Regulated Intervention: No

Study Description

Brief Summary: The purposes of the study are:

- To evaluate the effectiveness of a perceptual-based training program in enhancing cognitive and everyday functioning of older adults with mild cognitive impairment and mild dementia
- To evaluate the effectiveness of a semantic-based training program in enhancing cognitive and everyday functioning of older adults with mild cognitive impairment and mild dementia

Detailed Description: People with dementia experience a cognitive decline that affects their functional performance. It has been identified that encoding is less effective in people with dementia. Rehabilitating at the encoding stage is, therefore, essential. A recent systematic review found that effective interventions needed to include training in individually selected memory encoding strategies. These strategies are classified as either perceptual (visual) or semantic (association) memory encoding strategies.

Based on the above literature, the chief investigator (Associate Professor Karen Liu) ran a pilot study on an integrated home and community centre cognitive training programme for 20 elderly people with memory problem using a pre-test and post-test comparison design.

Extending on the positive results, the aim of this project was to investigate the effectiveness of perceptual and semantic encoding strategies (and compare with a control intervention). It also aims to promote the understanding of memory loss and dementia and the need for early intervention for family members of the participants.

Conditions

Conditions: Mild Cognitive Impairment
Mild Dementia

Keywords:

Study Design

Study Type: Interventional

Primary Purpose: Treatment

Study Phase: N/A

Interventional Study Model: Parallel Assignment

Number of Arms: 3

Masking: Double (Participant, Outcomes Assessor)

Allocation: Non-Randomized

Enrollment: 60 [Actual]

Arms and Interventions

Arms	Assigned Interventions
Experimental: Behavioral: perceptual-based memory encoding training<br>Participants receive perceptual-based memory encoding training	Behavioral: perceptual-based memory encoding training<br>Participants are trained in the use of perceptual-based memory encoding strategies, inclusive of visual imagery and the method of loci. Visual imagery involves the creation and encoding of mental images, while the method of loci refers to linking these images with specific places to facilitate memory (Simon et al., 2012). Participants are taught to visualise performing each step of a task in a familiar environment, such as in their home (Liu et al., 2009). After participants can correctly visualise the entire task, they then progress to actually performing the task. During the intervention sessions, 2-3 tasks are covered each session, and these tasks become progressively difficult as the weeks progress. The intervention lasts for 10 weeks with one session every week. Each session runs for 90 minutes. A follow-up home training program is run once a week for 30 minutes with the participant and their care-giver.
Experimental: Behavioral: Semantic-based memory encoding training<br>Participants receive semantic-based memory encoding training	Behavioral: semantic-based memory encoding training<br>Participants are asked to encode the steps of a task by forming an association of the steps and sequence. Participants are trained in the use of the chunking association method and honeycomb concept and perform different tasks each week, implementing this encoding strategy (Lim et al., 2012). The chunking association method breaks down information into smaller parts and helps with the encoding and retrieval of information. The honeycomb concept allows the steps to form a story in relation to place, time, characters, problem and solution and the story is verbalised. Participants then complete the task. During the intervention sessions, 2-3 tasks are covered each session, and these tasks become progressively difficult as the weeks progress. The intervention lasts for 10 weeks with one session every week. Each session runs for 90 minutes. A follow-up home training program is run once a week for 30 minutes with the participant and their care-giver.
Active Comparator: Behavioral : control group<br>Participants receive cognitive stimulation intervention	Behavioral: Cognitive stimulation group (control group)<br>Participants receiving cognitive stimulation intervention. It consists of eight sessions that train participants' visual attention and memory, auditory attention and memory and their application in daily activities. Two sessions are also given which allow for participants to apply the training practically in their daily lives. The intervention lasts for 10 weeks with one session every week. Each session runs for 90 minutes. A follow-up home training program is run once a week for 30 minutes with the participant and their care-giver.<br>Other Names:<br>• Cognitive stimulation group

Outcome Measures

Primary Outcome Measure:

1. Change in Disability Assessment for Dementia
   Change in Disability Assessment for Dementia to assess assistance required in basic daily tasks such as dressing, bathing

   [Time Frame: Within two days before the intervention and within two days after the 10-week intervention]
2. Change in Instrumental Activities of Daily Living Scale
   Change in Instrumental Activities of Daily Living Scale to assess assistance required in instrumental daily tasks such as laundry, meal preparation

   [Time Frame: Within two days before the intervention and within two days after the 10-week intervention]

Secondary Outcome Measure:

3. Change in Cognistat
   Change in Cognistat to assess general cognitive function

   [Time Frame: Within two days before the intervention and within two days after the 10-week intervention]
4. Change in Digit Span Test
   Change in Digit Span Test to assess attention and working memory

   [Time Frame: Within two days before the intervention and within two days after the 10-week intervention]
5. Change in Consortium to Establish a Registry for Alzheimer's Disease
   Change in Consortium to Establish a Registry for Alzheimer's Disease to assess cognitive function in verbal fluency, memory

   [Time Frame: Within two days before the intervention and within two days after the 10-week intervention]

Eligibility

Minimum Age: 60 Years

Maximum Age:

Sex: All

Gender Based:

Accepts Healthy Volunteers: No

Criteria: Selection Criteria:

- are aged 60 or above;
- have no previous psychiatric or memory disorder history or other neurological illness;
- have Mini-mental State Examination score (MMSE) greater than or equal to 21;
- have Clinical Dementia Rating score (CDR) of 0.5 or 1 indicating very mild and mild dementia;
- do not show sign of depression with score below 9 out of 30 in the Geriatric Depression Scale;
- are able to communicate effectively;
- have family member who can participate in the study; and
- voluntarily consent to participate in the study.

Contacts/Locations

Central Contact Person:

Central Contact Backup:

Study Officials:

Locations: **Australia, NSW**
Western Sydney University
Penrith, NSW, Australia, 2751
Contact: Karen P.Y. Liu, PhD +61 2 46203432
karen.liu@westernsydney.edu.au

Hong Kong
The Hong Kong Polytechnic University
Hong Kong, Hong Kong
Contact: Karen P.Y. Liu, PhD +61 2 46203432
karen.liu@westernsydney.edu.au

IPDSharing

Plan to Share IPD: No

References

Citations:

Links:

Available IPD/Information:

U.S. National Library of Medicine | U.S. National Institutes of Health | U.S. Department of Health & Human Services
