# Supplementary material for: Semantic-based memory-encoding strategy and cognitive stimulation in enhancing cognitive function and daily task performance for older adults with mild cognitive impairment: A pilot non-randomised cluster controlled trial
Source: PLoS One. 2023 Mar 27;18(3):e0283449. doi: 10.1371/journal.pone.0283449 (PMC10042350; doi:10.1371/journal.pone.0283449)
Supplement: S2 File — (PDF) [file pone.0283449.s003.pdf]

<u>Summary of ethics application</u>

Memory encoding strategies to promote healthy functioning in people with very mild and mild dementia: a clinical trial

Research plan

As part of the normal ageing process, older adults may experience deterioration in cognitive function that affects their independence in day-to-day functional activities. This impact on functional performance is even more people with dementia evident in.

There are three main stages in the formation and retrieval of memory. They are encoding, storage and retrieval. Encoding involves the formation of new memory traces. Storage refers to the maintenance of memory traces over time, and subsequent retrieval refers to processes that allow the access of stored memory information. In healthy older adults, cognitive training and stimulation can be used to maintain cognitive function (Ball et al., 2002). Most of the management approaches for patients with dementia is to provide remediation strategies to help in their retrieval. However, it has been identified that encoding is less effective in people with dementia (Belleville, Sylvain-Roy, de Boysson, & Menard, 2008). This could be due to their inability to organize information to be perceived. Rehabilitating at the encoding stage is therefore essential to solve their memory problem. Training approaches that are individually tailored for patients, based on their style of memory encoding, need to be used. This is supported by a recent systematic review on the effectiveness of different types of cognitive interventions (Jean, Bergeron, Thivierge, & Simard, 2010).

The review demonstrated that effective interventions needed to include training in individually selected memory encoding strategies. These strategies are classified as either perceptual memory encoding or semantic memory encoding strategies (Wu, Liu, & Chan, 2006). Jean et al. (2010) highlighted the limitations of completing such interventions in clinical environments. Instead, in order to promote its effectiveness and use by the general public, memory encoding interventions should be completed in the community. This enables input and involvement of family members which has been demonstrated to aid their understanding of dementia (Callahan & Hendrie, 2010).

Based on the above literature, the chief investigator (Associate Professor Karen Liu) ran a pilot study on an integrated home and community centre cognitive training programme for 20 elderly people with memory problem using a pre-test and post-test comparison design (Lim et al., 2012). The program consisted of 10 professionally-led sessions across 10 weeks. In addition to these sessions, trained family members and volunteers ran 20 home training sessions that were a continuation of the professionally-led cognition training sessions. Family members joined in the training with the people with memory problem and were taught how to enhance participants' engagement in the training activities during their day. After the intervention elements of cognition were examined using standardised assessments. The participants showed improved general attention ($p = 0.03$), memory ($p = 0.03$) and cognitive function including naming ($p = 0.02$), construction ($p = 0.01$), memory ($p = 0.02$) and similarities ($p = 0.001$necessary for their advanced daily functioning.

Aims of the research and the research question and/or hypotheses

Extending on the positive results, the aim of this project is to investigate the effectiveness of perceptual versus semantic encoding strategies, compare with a control group (that runs chess games, exercises and art and craft activities). This trial will allow investigation of the effectiveness of perceptual and semantic memory encoding strategies and produce evidence to enhance memory encoding and to benefit older adults with very mild and mild dementia. The study involves family members in the training and will also explore whether this training will have extra benefits on their understanding of dementia care.

Existing evidence shows that an eclectic approach, including training to stimulate attention, memory, abstract thinking, constructional ability, enhances cognitive function of people with dementia (Jean, et al., 2010). Our previous study on a multifaceted cognitive training that includes cognitive stimulation training and perceptual- and semantic-based memory strategies was proven to be effective (Lim, et al., 2012). However, these interventions provided a variety of stimulations and it was not certain which one actually worked for people with dementia. Our previous findings on the neural processes using event-related potentials revealed different neural mechanisms in tasks using perceptual-based and semantic-based strategy (Kuo, et al., 2012). Hudon and colleagues (2011) reported elderly people might benefit from semantic-based encoding. However, Froger and colleagues (2009) suggested that dementia sufferers could only benefit from semantic encoding with retrieval support. Our other study (Kuo, 2012) indicated that people with dementia took the advantage of the perceptual-based memory encoding strategy, but not the semantic-based one.
Based on these findings, our project lays the foundation for a theory-driven intervention that teases out the effect of unique cogntive profile of people of dementia to the use of memory encoding strategy in promoting cognitive function related to daily task performance.

Explain how the likely benefit of the research justifies the risks of harm or discomfort to participants.
The assessment included in this study may prove challenging for some participants given their cognitive impairment. Participants are given a list of support service in case they experience any potential anxiety or distress. The information is provided in the information sheet.
There is no other risk of harm or discomfort to participants other than their time commitment involved in the intervention. The offer of the memory intervention will add a benefit to the group by possibly enhancing their cognitive function. The involvement of family members will promote the understanding of memory loss and dementia and the need for early intervention for family members of the participants.

Are there any other risks involved in this research? eg. to the research team, the organisation, others.
What are these risks?
The study is conducted at the UnitingCare Aging. Potential risks could include safety of the participants such as fall risk.

Explain how these risks will be negated/minimised/managed.
The safety guideline specified by UnitingCare Aging will be followed to minimise the risk.

Explain how these risks will be monitored.
The procedure to monitor the risk as stated by UnitingCare Aging will be followed.

Explain how any harm to participants, resulting from these risks, will be reported.
If any harm to participants occur, it will be reported to the UnitingCare Aging in accordance with injury reporting procedures.
For the research personnel and honours students, it will be reported through the Injury Management System at the UWS.

Provide a concise detailed description, in not more than 200 words, in terms which are easily understood by the lay reader of what the participation will involve.
Participants will be invited to participate in a 10-week intervention programme for memory/cognitive/activity-based training. The program consists of:
1. Group training sessions: every week for 1 hour
2. Home-based training sessions: 2 sessions each week, each session lasting about 20 minutes.
Participants' partners or family members are invited to deliver the sessions. Training will be provided on how to conduct each home-based training session.
3. Participants and their partners / family members will receive information on memory and cognitive function of ageing and ways to help them maintain daily functioning. Additionally, information on dementia will also be provided.
Before and after the intervention programme, participants will be required to complete some assessments of everyday functioning and of cognition (thinking and memory skills).

Recruitment

What processes will be used to identify potential participants?

Talks and flyer will be distributed to aged care centres to advertise the project for participants recruitment.

Is it proposed to 'screen' or assess the suitability of the potential participants for

the study?

Yes

How will this be done?

Selection criteria are included in the flyer for advertisement of the project. Potential participants are then screened by the research team for suitability.

They are screened by the research personnel using the following tests:

- the Mini-mental State Examination score (MMSE);

- Clinical Dementia Rating score (CDR) ;

- the Geriatric Depression Scale.

Describe how initial contact will be made with potential participants.

Talks and flyer will be distributed to aged care centres to advertise the project for participants recruitment.

Those who are interested will be contacted in person.

Do you intend to include both males and females in this study? Yes

Is an advertisement, e-mail, website, letter or telephone call proposed as the form of initial contact with potential participants?

Yes

Provide details and a copy of text/script.

As we get older, we may experience some loss of thinking skills and/or memory complaints (cognitive decline) and may have or dementia. Early intervention (or treatment) is important to minimise the effect of memory decline on day-to-day functioning.

You are invited to participate in a new research study exploring the effectiveness of a specific memory training program in older adults to lessen the impact of memory loss in daily living. The study involves family members in the training. Their understanding on dementia care will also be evaluated.

Aims of the project

• To investigate the effectiveness of memory training on daily activities using two memory encoding strategies (visual or association) and activity-based training for people with mild dementia.

Project Content

Participants will be invited to participate in a 10-week intervention programme for memory/cognitive/activity-based training. The program consists of:

1. Group training sessions: every week for 1 hour

2. Home-based training sessions: 2 sessions each week, each session lasting about 20 minutes.

Participants' partners or family members are invited to deliver the sessions. Training will be provided on how to conduct each home-based training session.

3. Participants and their partners / family members will receive information on memory and cognitive function of ageing and ways to help them maintain daily functioning. Additionally, information on dementia will also be provided.

Before and after the intervention programme, participants will be required to complete some assessments of everyday functioning and of cognition (thinking and memory skills).

Who Can Join The Study?

You are invited to participate if you are:1) Aged 60 or above;

2) Have no previous psychiatric or memory disorder history or other neurological illness;

3) Have mild or moderate memory loss which can interefere a little with everyday activities. You may have been given a diagnosis of Mild Cognitive Impairment or mild dementia from your doctor;

4) Are able to communicate effectively; and

5) Have a partner or family member who can be involved in providing the home-based training for you

Please note that even if you agree to join the study, you are free to withdraw from the study at any time.

For more information, please contact Dr. Karen Liu, Associate Professor of Occupational Therapy at the University of Western Sydney on 02 4620 3432.

[Project investigators: Dr. Karen Liu, Ms. Michelle Bissett and Dr. Rosalind Bye, University of Western Sydney; Ms. Jacqueline Wesson and Dr. Melissa Slavin, Dementia Collaborative Research Centre]

Consent process

Will consent for participation in this research be sought from all participants? Yes

Will there be participants who have capacity to give consent for themselves? Yes

What mechanisms/assessments/tools are to be used, if any, to determine each of these participant's capacity to decide whether or not to participate?

The research participants' rights as specified in the Alzheimer's Australia will be followed.

In addition, the process of obtaining informed consent involves determining whether a person has the necessary competence.

According to the Mental Capacity Act in UK (2005), the assessment of competence involves paying attention to four main abilities. The researcher will make sure the potential participant has all these abilities.

1. the person must have sufficient capacity to understand the information;

2. the person must be able to understand what the decision is about, why they are being asked to make it, and what the consequences of making or not making that decision might be;

3. the participants understand the possible benefits, risks and inconvenience linked to participating in research;

4. the participants must have the ability to communicate their decision.

All potential participants are invited or referred by the UnitingCare Aging to attend the information sessions.

After the information session, they are invited to give their name and contact information if they are interested to know more or participate in the study. They are then screened by the following criteria and methods.

1. Age 60 or above is determined by their photo ID.

2. They will be reported to have no previous psychiatric or memory disorder history or other neurological illness.

3. They have Mini-mental State Examination score (MMSE) greater than or equal to 21.

4. They will be assessed with Clinical Dementia Rating score (CDR) of 0.5 or 1 indicating very mild and mild dementia.

5. They are assessed with Geriatric Depression Scale with score below 9 out of 30 indicating no sign of depression;

6. They are able to communicate effectively.

The following tests will be used:

- the Mini-mental State Examination score (MMSE);

- Clinical Dementia Rating score (CDR) ;

- the Geriatric Depression Scale.

Their partner / family members will have reported to be healthy, and able to communicate effectively.

Describe the consent process, ie how participants or those deciding for them will be informed about, and choose whether or not to participate in, the project.

After fulfiling the selection criteria, participants and their partner / family members will be explained on the study details. Written information sheet will be delivered to potential participants and their partner / family members. If they agree to participate after receiving both verbal and written information, informed written consent will be obtained from both the participants and their partner / family member before the beginning of the study. In case the partner / family member does not consent to participate, the participant will not be recruited for the study.

All participants are free to withdraw from the study at any time, or re-evaluation of the consent will take place in week 5 of the study.

If a participant or person on behalf of a participant chooses not to participate, are there specific consequences of which they should be made aware, prior to making this decision?

No.

In case either the partner / family member or participant does not consent to participate, the participant and partner/family will not be recruited for the study.

The consents from both parties are collected separately to ensure they do not coerce each other to participate.

Might individual participants be identifiable by other members of their group, and if so could this identification expose them to risks?

The carers/families may be known and identifiable to each other. This would not expose participants to risk as the personal information of the participants is kept confidential.

If a participant or person on behalf of a participant chooses to withdraw from the research, are there specific consequences of which they should be made aware, prior to giving consent?

No.

Specify the nature and value of any proposed incentive/payment (eg. movie tickets, food vouchers) or reimbursement (eg travel expenses) to participants.

There is no payment involved.

Explain why this offer will not impair the voluntary nature of the consent, whether by participants' or persons deciding for their behalf.

As there is no payment involved, the participation is completely voluntary.

Do you propose to obtain consent from individual participants for your use of their stored data/samples for this research project?

No

Give justification

Data gather from the current study will be used. No data is required from previous data bases.
