# Supplementary material for: Semantic-based memory-encoding strategy and cognitive stimulation in enhancing cognitive function and daily task performance for older adults with mild cognitive impairment: A pilot non-randomised cluster controlled trial
Source: PLoS One. 2023 Mar 27;18(3):e0283449. doi: 10.1371/journal.pone.0283449 (PMC10042350; doi:10.1371/journal.pone.0283449)
Supplement: S1 Appendix — (DOCX) [file pone.0283449.s004.docx]

## Appendix

**Example practice of the semantic-based memory-encoding strategy intervention**

| **Task** |
| --- |
| Introduce the strategies:   - Chunking:   - Chunking is useful in organising information into small and manageable steps for easy encoding. - Honeycomb association method:   - Helps to map the problem into the relationship of objects, the time of doing the tasks, the place of doing it and the person who is involved in the tasks. This will help the patients encode better to form semantic meaning of the tasks to be remembered. - To make use of both strategies to recall the steps from each task. - Participants have to say aloud the steps involved. - Emphasize on learning to use the strategies. |
| **Example task: Washing the dishes**   1. To wash 10 to 15 dishes. 2. Put them onto the rack for drying after washing. 3. This activity is done standing; participants may request for a chair to sit down if needed.   **Procedures:**   1. Participants to tell the steps of the tasks (chunking). 2. Participants to associate the tasks to objects, place, time and persons (honeycomb concept).  \| **Objects** \| - Dirty dishes - Dishwashing liquid - Dish rack \| \| --- \| --- \| \| **Place** \| - Kitchen - Standing at the sink \| \| **Time** \| - After a meal \| \| **Persons** \| - Participants themselves and/or family members \| \| **Solution** \| - Put all the dishes into the sink and arrange them properly in accordance with the participants’ preferences - Turn on the water tap, run the water onto the dishes - Put some soap onto the sponge - Soap the dirty dishes - Wash away the soap - Put the clean dishes on the rack for drying. \|  1. Participants to repeat the associations. 2. Participants to say aloud all the steps. 3. Participants to associate the steps of a task by:    - - relationship of the objects      - time of doing the tasks      - place of doing the step      - person(s) doing the tasks 4. Each participant to take turns in saying aloud the associations so that the step in each task is connected. 5. Participants to practise the tasks. |
